# Microbiological Advantages of Open Incisional Biopsies for the Diagnosis of Suspected Periprosthetic Joint Infections

**DOI:** 10.3390/jcm11102730

**Published:** 2022-05-12

**Authors:** Marcel Niemann, Ellen Otto, Karl F. Braun, Frank Graef, Sufian S. Ahmad, Sebastian Hardt, Ulrich Stöckle, Andrej Trampuz, Sebastian Meller

**Affiliations:** 1 Center for Musculoskeletal Surgery, Charité—Universitätsmedizin Berlin, Corporate Member of Freie Universität Berlin, Humboldt-Universität zu Berlin, and Berlin Institute of Health, 13353 Berlin, Germany; ellen.otto@charite.de (E.O.); karl.braun@charite.de (K.F.B.); frank.graef@charite.de (F.G.); sufian@ahmadortho.com (S.S.A.); sebastian.hardt@charite.de (S.H.); ulrich.stoeckle@charite.de (U.S.); andrej.trampuz@charite.de (A.T.); sebastian.meller@charite.de (S.M.); 2 Julius Wolff Institute for Biomechanics and Musculoskeletal Regeneration, Berlin Institute of Health at Charité—Universitätsmedizin Berlin, 13353 Berlin, Germany; 3 Department of Trauma Surgery, University Hospital Rechts der Isar, Technical University of Munich, 80333 Munich, Germany; 4 Department of Orthopedic Surgery, Hannover Medical School, 30625 Hannover, Germany

**Keywords:** periprosthetic joint infection, total hip arthroplasty, total knee arthroplasty, individual medicine, microbiology, diagnostics

## Abstract

Background: Periprosthetic joint infection (PJI) represents a serious complication following total hip (THA) and knee arthroplasty (TKA). When preoperative synovial fluid cultures remain inconclusive, open incisional joint biopsy (OIB) can support causative microorganism identification. Objective: This study investigates the potential benefit of OIB in THA and TKA patients with suspected PJI and ambigious diagnostic results following synovial fluid aspiration. Methods: We retrospectively assessed all patients treated from 2016 to 2020 with suspected PJI. Comparing the microbiology of OIB and the following revision surgery, we calculated sensitivity, specificity, positive predictive value (PPV), negative predictive value (NPV), and the number needed to treat (NNT). Results: We examined the diagnostic validity of OIB in 38 patients (20 female) with a median age of 66.5 years. In THA patients (*n* = 10), sensitivity was 75%, specificity was 66.67%, PPV was 60%, NPV was 80%, and NNT was 2.5. In TKA patients (*n* = 28), sensitivity was 62.5%, specificity was 95.24%, PPV was 83.33%, NPV was 86.96%, and NNT was 1.42. Conclusions: Our results indicate that OIB represents an adequate diagnostic tool when previously assessed microbiological results remain inconclusive. Particularly in TKA patients, OIB showed an exceptionally high specificity, PPV, and NPV, whereas the predictive validity of the diagnosis of PJI in THA patients remained low.

## 1. Introduction

Periprosthetic joint infection (PJI) represents a serious complication [1,2,3] opposed to the outstanding long-term results of total hip (THA) and knee arthroplasty (TKA) [4,5]. PJI is the most common cause of arthroplasty failure due to component loosening and subsequent instability [6]. While the overall incidence of PJI in THA and TKA is merely around 2% [7,8], affected patients experience an impaired quality of life. This may last for years, even after successful surgical treatment [9,10]. Accordingly, a preference for an early and accurate diagnosis of PJI is one of the most accepted consensuses in orthopaedic surgery [11].

Still, PJI poses a major clinical challenge [12,13,14], demanding standardized and multidisciplinary diagnostic and surgical treatment algorithms [15]. The diagnostic protocol for suspected PJI comprises the recent medical history of patients, clinical signs, and a laboratory as well as a microbiological work-up of blood, synovial fluid, and deep tissue samples [12,15,16]. In recent years, these diagnostic tools were supplemented with sonication, which once more improved the diagnostic accuracy when PJI is suspected [17]. However, up to 42% of all synovial fluid and tissue samples sent to microbiology are culture-negative [7,18,19], thus requiring particularly dedicated treatment-strategy planning.

In acute PJI with reduced bone and soft tissue quality, instability, or difficult-to-treat infections as well as in chronic PJI, the complete exchange of the prosthesis is recommended [16,20]. Although two-stage revision concepts ensure satisfactory long-term results [21,22,23], one-stage revision approaches are of particular interest [24,25] due to reduced functional impairment and mortality [23,26]. Overall, treatment failure for re-infection rates following arthroplasty exchange ranges between 10% and 25% [27,28,29]. For years, most authors attributed the sufficient treatment of PJI solely to multiple-stage revision approaches [18,19,27,30]. However, recent data prove satisfactory treatment results following single-stage revision surgery as long as patients are adequately selected for eligibility [28,31]. These single-stage approaches offer some advantages, such as reduced surgery frequency and shortened hospitalization [31], which can help to lower the economic burden related to revision surgeries [8,25]. However, the preoperative identification of causal microorganisms is of utmost importance for properly choosing the most appropriate therapeutic approach [31].

In order to enhance causative microorganism identification, open incisional joint biopsy (OIB) sampling of periprosthetic and synovial tissue can be considered when previous diagnostics remain inconclusive [15] or are not feasible due to dry tap aspirations. Although the role of OIB is currently discussed [32,33,34], fundamental clinical data remain limited. Therefore, this study aims to provide further evidence on the potential benefit of OIB in cases of inconclusive microbiological results in suspected PJI of THA and TKA.

## 2. Methods

Once local ethics committee approval was obtained (application number EA4/040/14), we retrospectively assessed all patients treated at one center for suspected PJI between January 2016 and December 2020. Patients’ data extracted from the electronic medical data system SAP (SAP ERP 6.0 EHP4, SAP AG, Walldorf, Germany) were age, gender, the American Society of Anesthesiologists physical status classification system (ASA), a summary of the medical history through the Charlson comorbidity index (CCI) [35], the primary implantation date of the arthroplasties, and laboratory along with microbiological results.

At our study center, the Musculoskeletal Infection Society (MSIS) criteria [36] and the updated standard diagnostic protocol recommended by the PRO-IMPLANT Foundation [15] were applied for the diagnosis of suspected PJI. These demand the aspiration of synovial fluids (following an antibiotic-free interval of at least 14 days) and whole blood samples, which are sent for laboratory and microbiological testing, including incubation for 14 days. Leukocyte count, proportion of polymorphonuclear leukocytes (PMN), germ growth in synovial fluid samples, leukocyte (normal range 3.9–10.5/nL), and c-reactive protein (CRP, normal range < 5 mg/L) concentrations in the whole blood samples were assessed. In synovial fluids, leukocyte counts over 2/nL along with a portion of PMN over 70% were considered suspicious for PJI. The final microbiological results were discussed with infectious disease specialists. Any germ growths below 50 colony-forming units (CFU) in only one of the samples were scored as contaminations and excluded from further analysis.

OIB was performed in patients with previously inconclusive (MSIS of two to five) or insufficient diagnostic results (predominantly following dry tap synovial fluid aspirations). The major aim of OIB was to most accurately predict the microbiological results of patients following arthroplasty revision surgery. OIB was performed under general anaesthisia by using dry arthroscopy or a small incision using the pre-existing surgical approach from primary arthroplasty. In addition to synovial fluid, at least five deep tissue samples of representative regions in contact with the arthroplasty were collected for microbiological diagnostics.

In definite revision surgery, representative deep tissue samples were acquired as well. Any exchanged arthroplasty components were sent for sonication [17].

Statistical analysis was performed using GraphPad Prism (GraphPad Prism for macOS, Version 9.3.1, GraphPad Holdings, LLC, San Diego, CA, USA). Data distribution was tested using histograms and Q-Q plots. We calculated sensitivity, specificity, positive predictive values (PPV), negative predictive values (NPV), and number needed to treat (NNT) for OIB in the diagnostic cascade of suspected PJI. Independent samples were assessed by conducting a Mann–Whitney *U* test and dependent samples were evaluated by applying a Wilcoxon signed-rank test. Unless stated otherwise, frequencies are displayed as numbers (%) and not normally distributed values as median (interquartile range (IQR)). All *p*-values are two-tailed, and *p*-values ≤ 0.05 were considered statistically significant.

## 3. Results

Between January 2016 and December 2020, 38 patients (20 (52.36%) female) received an OIB due to suspected PJI at our center. Of these, 10 patients had a THA, and 28 had a TKA. The median time since index primary joint replacement was 126 months (IQR 40.75–172.5) in THA and 24 months (IQR 13–71) in TKA. Two patients (20%) following THA and 21 (75%) of the TKA patients had no revision surgeries following the primary joint replacement. All other patients in the THA and TKA group had a median of 1 (IQR 1–2) and 1.5 (IQR 1–2.75) revision surgeries following the initial implantation, respectively. The median time since last revision surgery was 19.5 months (IQR 15–95.25) in THA and 10 months (IQR 6–33) in TKA patients. The demographic characteristics of the study cohort are displayed in Table 1.

### 3.1. Diagnostic Approach in Patients Suspected for PJI

All patients solely declared pain in the affected joint. None of these patients were examined for PJI due to a persistent wound secretion following primary or revision arthroplasty.

In the total cohort, median CRP was 4.5 mg/L (IQR 2.15–12.5 mg/L) and median leukocyte count was 8.02/nL (IQR 6.89–9.14/nL). In THA patiens, CRP was 3.15 mg/L (IQR 1.05–9.55 mg/L) and leukocyte count was 7.64/nL (IQR 6.41–8.89/nL). In TKA patients, both CRP (5 mg/L, IQR 2.6–12.7 mg/L, *p* = 0.27) and leukocyte count (8.27/nL, IQR 7.04–9.36/nL, *p* = 0.48) were not significantly higher when compared to THA patients.

In twelve (31.58%) patients, synovial fluid aspiration was successfully achieved in our outpatient clinic. The median leukocyte count was 2.42/nL (IQR 0.56–4.76/nL) and median portion of PMN was 45.59% (IQR 26.95–75.55%). Eleven (28.95%) aspiration samples were sent for microbiological testing, and in none of these samples was germ growth detected.

**Figure 1:**
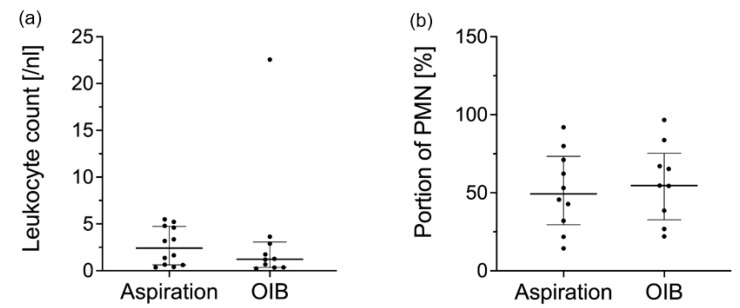
Laboratory results of synovial fluid aspiration in the outpatient setting and during OIB. (**a**) Leukocyte count and (**b**) proportion of PMN in the synovial fluid aspirations are displayed as scatter dot plot presenting medians and IQR. There were no significant differences between values assessed with outpatient and synovial fluid aspirations from OIB. This visualization solely displays patients with available outpatient and synovial fluid aspiration results from OIB (**a**) *n* = 10, (**b**) *n* = 9. Abbreviations: PMN: polymorphonuclear leukocytes; OIB: open incisional joint biopsy; IQR: Interquartile range.

Therefore, patients were admitted for supplementary OIB. All patients of THA and one (3.57%) of the TKA group received OIB through the surgical approach previously chosen for primary arthroplasty. The other 27 (96.43%) patients with TKA received OIB through arthroscopy. OIB took place 31 days (IQR 24–83 days) after the aforementioned outpatient synovial fluid aspiration. The synovial fluids assessed through OIB revealed a median leukocyte count of 1.72/nL (IQR 0.36–5.21/nL) and a median portion of PMN of 54.59% (IQR 30.99–87.06%). Median leukocyte counts did not differ (*p* = 0.23) between THA (3.62/nL, IQR 0.84–13.99/nL) and TKA patients (1.27/nL, IQR 0.29–4.91/nL). The median portions of PMN were not different (*p* = 0.13) between THA (64.85%, IQR 45.68–87.2%) and TKA patients (52.21%, IQR 29.25–86.32%). We did not observe any local wound problems following OIB. Figure 1 displays the laboratory results of synovial fluid aspiration.

According to the aforementioned diagnostic results, we decided on five (50%) and 17 (60.71%) one-stage revision surgeries in the THA and TKA group, respectively. Further, five (50%) and eleven (39.29%) patients received two-stage revision surgery following THA and TKA, respectively. In most samples collected at OIB and during revision surgery, no germ growth was detected. The most commonly identified specimens were coagulase-negative *Staphylococci*. Table 2 displays microbiological findings that compare OIB and revision surgeries.

The median follow-up of the cohort was 14 months (IQR 7–33 months). The median follow-up duration was 8 months (IQR 5.5–17 months) and 22 months (IQR 8–40 months) in THA and TKA patients, respectively. During that period, three (7.9%) patients (one (10%) in THA and two (7.14%) in the TKA group) needed further revisions: The first patient had one-stage revision with no detectable germ growth in any sample and needed aseptic component exchange due to patellar clunk syndrome later on. The second patient had two-stage revision surgery with detected coagulase-negative *Staphylococci* in microbiological samples and needed local wound revision two weeks afterward due to persistent wound drainage. The patient received debridement, antibiotics, and implant retention (DAIR) without detectable germ growth in any sample and did not need further revision surgeries. The third patient had one-stage revision surgery and needed another component exchange due to PJI ten months following OIB. Microbiological samples from the revision surgery detected a coagulase-negative staphylococci, while the previous OIB had not shown any germ growth or laboratory abnormalities (synovial fluid leukocyte count of 1.74/nL with a proportion of 38.6% of PMN, CRP 5 mg/L, leukocyte count could not be measured).

### 3.2. Diagnostic Quality Assessment of OIB

In all patients, the sensitivity of OIB was 66.67% (95% CI 39.06–86.19) and specificity was 88.89% (95% CI 71.94–96.15), resulting in a PPV of 72.73% (95% CI 43.44–90.25), an NPV of 85.71% (95% CI 68.51–94.3), and an NNT of 1.71 (95% CI 1.24–5.03).

Considering only THA patients, the sensitivity of OIB was 75% (95% CI 30.06–98.72) and specificity was 66.67% (95% CI 30–94.08), which led to a PPV of 60% (95% CI 23.07–92.89), an NPV of 80% (95% CI 37.55–98.97), and an NNT of 2.5 (95% CI not computable).

In TKA patients, the sensitivity of OIB was lower (62.5%, 95% CI 30.57–86.32), while specificity was higher (95.24%, 95% CI 77.33–99.76) when compared to THA patients. Furthermore, PPV (83.33%, 95% CI 43.65–99.15) and NPV (86.96%, 95% CI 67.87–95.46) were higher, respectively, and NNT was lower (1.42, 95% CI 1.13–5.35) when compared to THA patients.

## 4. Discussion

In the present study, we assessed the diagnostic validity of OIB for suspected PJI and inconclusive diagnostic results. Overall, OIB showed moderate sensitivity but high specificity after synovial fluid aspiration, with a particularly high predictive diagnostic quality in TKA patients. While sensitivity was slightly higher in THA patients, specificity and NPV were markedly lower, which implies a potential risk of false negative assumptions following OIB in these patients.

When PJI is suspected and conventional diagnostic approaches are inconclusive or not feasible (e.g., dry taps in synovial fluid aspiration attempts), OIB expands the spectrum of available diagnostic tools. A recent meta-analysis concluded that OIB is not superior to synovial fluid aspirations alone but contributes to the already existing diagnostic algorithms and improves diagnostic accuracy [34]. Since that analysis, few authors published results concerning the usage of OIB for the diagnosis of PJI [32,33]. Klaber et al. assessed OIB as a diagnostic tool following two culture-negative aspirations in suspected PJI in THA and TKA [32]. In their cohort, sensitivity was 69.4%, specificity was 89.1%, PPV was 86%, and NPV was 75%, which is highly comparable with our data. In their sub-analysis of THA and TKA patients, however, diagnostic accuracy was markedly lower in TKA when compared to THA patients. These results are contrary to our observations, which showed a higher diagnostic predictability in TKA patients. A possible explanation for this may be the assessed endpoints, as Klaber et al. investigated the predictability of OIB for the diagnosis of PJI according to the MSIS criteria, while we assessed the predictability of the causative germ spectrum by comparing OIB and revision surgery. Schwarze et al. investigated the use of OIB in patients with TKA component loosening and inconclusive previous diagnostic results [33]. They observed a sensitivity of 47%, specificity of 77%, PPV of 39%, and NPV of 62%. These diagnostic accuracy measures were lower when compared to Schwarze et al.’s sub-analysis of TKA patients [32] and our results. This might be explained by the surgical approach applied. In the cohort of Schwarze et al. [33], supra-patellar mini-arthrotomy was adopted to collect samples, while we used an arthroscopic approach in all but one of the TKA patients. The latter approach potentially allows an easier sample collection in different representative regions when compared to mini-arthrotomies.

In our cohort, we observed unexpected positive cultures in 7.89% of the patients, of which one patient needed additional revision surgeries due to persisting PJI. That rate was lower when compared to data of previous authors who found unexpected positive cultures in 23% of all cases [33]. Revision surgeries related to unexpected cultures were not reported by the authors. Such unexpected positive results need to be monitored with particular caution, as patients may have an increased risk of persisting PJI [37].

In the present study, the most commonly detected germs were coagulase-negative staphylococci and *Cutibaterium* species, followed by *Staphylococcus* *aureus*. These results are in accordance with previous OIB results [32,33]. Furthermore, Schulz et al. observed a highly comparable germ spectrum in synovial fluid aspirations [38]. However, the overall concordance between preoperative synovial fluid aspirations and intraoperative tissue was as low as 52%, which is accompanied by the highly heterogeneous diagnostic accuracy measures reported across the literature [34]. This underlines the importance of continuously evaluating the potential advantage of supplementary diagnostic appoaches in suspected PJI, such as OIB.

When interpreting our results, the following limitations need to be considered. First, we provide a monocentric data analysis of patients with suspected PJI. This entails a potential bias caused by in-house standards, thereby limiting comparability. In the future, studies should be conducted in a multi-center setting. Furthermore, this analysis solely provides retrospective data, which potentially limits overall data quality. Future studies should be prospectively conducted. Second, the approach for OIB in THA and TKA differed. While all THA patients received OIB with the approach established during primary arthroplasty, most TKA patients received OIB through arthroscopy. As a result, the comparison of diagnostic validity of OIB between THA and TKA patients may be limited. This may be further limited by the small sample size and limited follow-up. In particular, sample size potentially limits stastistical results and should be taken into account when planning future studies. However, our results strengthen presumptions that OIB does not lead to equally reliable results in all patients. Furthermore, the performance of OIB implies an extra surgical procedure, including anesthesia, which can result in an additional risk for the patient and raises overall economic costs. Therefore, OIB should solely be used as a supplementary diagnostic approach for previously inconclusive diagnostic results.

## 5. Conclusions

In our study, OIB was observed to attain high specificity, PPV, and NPV, especially in TKA patients. This supports orthopaedic surgeons to certainly distinguish between aseptic and septic revision surgeries in advance. Therefore, the use of OIB in the diagnostic cascade of suspected PJI can support interdisciplinary decisions on the most adequate treatment strategy in situations of ambiguous joint aspiration results. However, the diagnostic limitations of OIB need to be kept in mind, especially for its usage in THA patients.

## Figures and Tables

**Table 1:** Demographic characteristics of the study cohort.

		Total (*n* = 38)	THA (*n* = 10)	TKA (*n* = 28)
age (years)		66.5 (IQR 59.75–73)	62 (IQR 46.75–68.25)	67 (IQR 61–74)
gender	female (%)	20 (52.63%)	2 (20%)	18 (64.29%)
	male (%)	18 (47.37%)	8 (80%)	10 (35.71%)
ASA		2 (IQR 2–3)	2 (IQR 1–2.25)	2 (IQR 2–3)
CCI		3 (IQR 2–4)	2 (IQR 0.5–3)	3 (IQR 2–4)
BMI		28.88 (IQR 26.42–34.88)	27.62 (IQR 25.17–34.03)	31.05 (IQR 26.71–35.48)

Abbreviations: THA: total hip arthroplasty; TKA: total knee arthroplasty; IQR: Interquartile range; ASA: American Society of Anesthesiologists physical status classification system; CCI: Charlson comorbidity index; BMI: body bass index.

**Table 2:** Microbiological results of the samples.

		Open Incicional Biopsy		Revision Surgery	
		THA (*n* = 10)	TKA (*n* = 28)	THA (*n* = 10)	TKA (*n* = 28)
High virulent pathogens	*Staphylococcus aureus*	0 (0%)	1 (3.45%)	1 (10%)	1 (3.45%)
	*Staphylococcus lugdunensis*	0 (0%)	1 (3.45%)	0 (0%)	0 (0%)
Low virulent pathogens	Coagulase-negative staphylococci	3 (30%)	5 (17.24%)	3 (30%)	5 (17.24%)
	*Cutibacterium* spp.	1 (10%)	0 (0%)	1 (10%)	0 (0%)
	*Enterococcus* spp.	0 (0%)	1 (3.45%)	0 (0%)	0 (0%)
No germ growth		6 (60%)	21 (72.41%)	5 (50%)	23 (79.31%)

Abbreviations: TKA: total knee arthroplasty; THA: total hip arthroplasty; spp.: Species.

## Data Availability

The data presented in this study are available upon request from the corresponding author. The data are not publicly available due to regulations of the local institutional ethics board.
